# Relationship between employment histories and frailty trajectories in later life: evidence from the English Longitudinal Study of Ageing

**DOI:** 10.1136/jech-2016-207887

**Published:** 2016-12-02

**Authors:** Wentian Lu, Rebecca Benson, Karen Glaser, Loretta G Platts, Laurie M Corna, Diana Worts, Peggy McDonough, Giorgio Di Gessa, Debora Price, Amanda Sacker

**Affiliations:** 1 ESRC International Centre for Lifecourse Studies in Society and Health (ICLS), Research Department of Epidemiology and Public Health, Institute of Epidemiology and Health Care, University College London, London, UK; 2 Research Department of Epidemiology and Public Health, Institute of Epidemiology and Health Care, University College London, London, UK; 3 Department of Global Health & Social Medicine, Institute of Gerontology, School of Social Science and Public Policy, King's College London, London, UK; 4 Stress Research Institute, Stockholm University, Stockholm, Sweden; 5 Dalla Lana School of Public Health, University of Toronto, Toronto, Ontario, Canada; 6 Department of Social Policy, The London School of Economics and Political Science, London, UK; 7 Manchester Institute for Collaborative Research on Ageing, University of Manchester, Manchester, UK

**Keywords:** EMPLOYMENT, Social and life-course epidemiology, AGEING

## Abstract

**Background:**

Given the acceleration of population ageing and policy changes to extend working lives, evidence is needed on the ability of older adults to work for longer. To understand more about the health impacts of work, this study examined the relationship between employment histories before retirement and trajectories of frailty thereafter.

**Methods:**

The sample comprised 2765 women and 1621 men from the English Longitudinal Study of Ageing. We used gendered typologies of life-time employment and a frailty index (FI). Multilevel growth curve models were used to predict frailty trajectories by employment histories.

**Results:**

Women who had a short break for family care, then did part-time work till 59 years had a lower FI after 60 years than those who undertook full-time work until 59 years. Women who were largely family carers or non-employed throughout adulthood, had higher levels of frailty at 60 years but experienced a slower decline with age. Men who worked full-time but early exited at either 49 or 60 years had a higher FI at 65 years than those who worked full-time up to 65 years. Interaction between employment histories and age indicated that men in full-time work who experienced an early exit at 49 tended to report slower declines.

**Conclusions:**

For women, experiencing distinct periods throughout the lifecourse of either work or family care may be advantageous for lessening frailty risk in later life. For men, leaving paid employment before 65 years seems to be beneficial for decelerating increases in frailty thereafter. Continuous full-time work until retirement age conferred no long-term health benefits.

## Introduction

In 2010, 10 million people in the UK were 65 years and older and the number is estimated to nearly double to around 19 million by 2050.^1^ A range of recent employment policy initiatives are aimed at supporting older people to work for longer, potentially benefiting the national economy and improving individual savings for retirement.^2^ However, we know little about the implications of working beyond state pension age for health. Given policy changes that expect people to work for longer it is critical to investigate whether people's lifetime exposure to work affects later life health. Adverse physical and psychosocial working conditions contribute to physiological changes in the body leading to an increased risk of observable pathology.^3^ Lifetime exposure to work, summarised in employment history profiles, can help us understand more about the relationship between work and health declines in later life.

Many studies looked at physical or mental health using single or a limited selection of health indicators. Frailty is commonly considered to be a clinical syndrome among older adults associated with numerous poor health outcomes including morbidity, incident disability, hospitalisation, institutionalisation and death,^4–7^ and may be a better measure than just one indicator. In the UK the prevalence of frailty was found to be 8.5% in women and 4.1% in men aged 65–74 years old in 2010.^8^ It has been estimated that in 2020, one of the top three causes of death in the UK will be frailty/dementia.^9^ There is currently no universally accepted definition or model of frailty; however, general agreement has been reached that the causes are complex and likely to involve both biological and psychosocial mechanisms.^10^ ^11^ The two most commonly used models are the ‘Phenotype Model’ and the ‘Cumulative Deficit Model’. The first model operationalises frailty as a phenotype,^6^ and the second as physical decline or a non-specific vulnerability.^4^ The first conceptualisation of frailty includes five criteria: shrinking, weakness, exhaustion, slowness and low activity.^6^ The latter conceptualisation assumes an accumulation of a large number of deficits (eg, symptoms such as pain while walking; signs such as tremors; and diseases such as emphysema or dementia) that may occur with ageing and are combined to give a ‘frailty index (FI)’, with higher values on the FI indicating greater likelihood of frailty.^12^

A few studies have measured frailty in England on nationally representative survey populations,^7^ ^13^ ^14^ but none have investigated how paid employment histories before retirement are associated with frailty trajectories in later life. Exploring the relationship between employment histories and the development of frailty could inform the debate on the public health impacts of working longer. This study therefore seeks to add to the evidence by examining the relationship between employment histories before state pension age for women and men in the English Longitudinal Study of Ageing (ELSA) and frailty trajectories in later life. Given the discourse on work-life balance,^15^ ^16^ we take into account marital and fertility histories as well as other confounding and explanatory factors identified in prior research.

## Methods

### Participants

ELSA is a nationally representative panel survey based on community-dwelling older adults in England. It began in 2002 and has recruited 11 392 men and women aged 50 years or more to follow-up.^17^ The sample has been revisited every 2 years since 2002.^17^ Face-to-face interviews and self-completion assessments were carried out in all waves. Nurse visits were undertaken in waves 2 (2004/2005), 4 (2008/2009) and 6 (2012/2013), collecting anthropometric data and testing for physical performance.^17^ In wave 3, a life history interview was completed that captured retrospective information about employment; partnerships and children, as well as childhood circumstances and health. The present study used data from a subsample of 4386 ELSA respondents (1621 men and 2765 women) aged 60 (women) or 65 (men) to 90 years old in wave 2 who also completed the life history questionnaire in wave 3.

### Measures

#### Frailty

A FI is preferred over a frailty phenotype measure due to its finer graded risk scale, and robustness for clinical inference irrespective of the number and composition of its constituent items.^18–20^ A FI, similar to that developed in a previous study,^13^ captures 60 deficits covering a wide range of domains (ie, activities of daily living, cognition, chronic conditions, pain, depression, cardiovascular diseases, falls and fractures and joint replacement). See also online supplementary table S1. Each deficit was dichotomised or categorised into quartiles, averaged and then standardised to a 0–1 interval.^21^ The FI was derived for individuals who had non-missing items for at least 30 of the 60 deficits.^13^

10.1136/jech-2016-207887.supp1 supplementary table

#### Employment histories

The employment history data in wave 3 was used to derive individual histories of labour market involvement between the ages of 16 and 64 years for men, and 16 and 59 years for women. Individual economic activity was coded at each age, distinguishing between full-time employment, part-time employment (≤30 hours/week) and other activities (unemployment, provision of family care, incapacity, education and retirement). Missing information on work variables was first imputed using Halpin's method for estimation of missing sequence data.^22^ Twenty imputed data sets were created with employment histories from the filled-in sequences. The patterns of economic activity experienced by men and women over their lives were classified using optimal matching analysis, a type of sequence analysis which characterises progression throughout the life-course in a holistic manner.^23^ We used an ‘ideal type’ comparison method which compares all observed sequences of work events against a set of ideal type trajectories,^24^ as fully described in Corna *et al*.^25^ Our analysis included five ideal employment histories for men (employed full-time throughout; not employed throughout; full-time throughout until early exit at 60 or at 49 years; and late start of paid work and exit at 60 years) and seven for women (employed full-time throughout, not employed throughout, weak attachment to the labour market and early exit, long or short career break followed by part-time employment, family care to full-time work and full-time to part-time work) shown in table 1 (see also online supplementary figures S1 and S2).

**Table 1:** Distribution of employment histories by gender

Gender	Types	Description	%
Female	FTT	Full-time throughout	26.05
	NET	Non employment throughout/family carers	23.10
	WA	Weak attachment, early exit	5.99
	LCB	Family carer to part-time (longer career break)	13.23
	SCB	Family carer to part-time (short career break)	11.16
	FC→FT	Family care to full-time	16.29
	FT→PT	Full-time to part-time	4.17
Male	FTT	Full-time throughout	48.61
	NET	Non-employment throughout	4.11
	FTE49	Full-time very early exit (at 49)	9.20
	FTE60	Full-time early exit (at 60)	30.36
	LSE60	Late start, early exit (at 60)	7.72

10.1136/jech-2016-207887.supp4 supplementary figures

#### Covariates

Baseline covariates known to be associated with work and health were fathers' social class (at 14 years: non-manual, manual, unclassifiable);^26^ self-rated health during childhood (poor, fair, good, very good, excellent);^27^ ^28^ education (highest education qualification: 6-point scale from no qualification to NVQ5/Degree or equivalent); social class (RGSC: I-Professional to V-Unskilled manual); marital history (never married, long-term married, marriage ended early and marriage ended late);^15^ fertility history (no children, one child early, one child later, children early, children later and early large family).^16^ These marital and fertility histories were derived using the same optimal matching methodology employed for the employment histories (see Corna *et al*^25^ for further details).

The time varying covariates considered were: age and age^2^ (centred on 60/65), partnership (living with partner vs not), smoking status (non-smoker vs smoker), drinking (mean days per year), index of multiple deprivation (IMD, ranges from 0 to 9)^29^ ^30^ and non-pension wealth (quintiles from highest to lowest income, ranges from 0 to 4).^31^

### Statistical analysis

Multiple imputations were used for dealing with missing values in the main exposure and other covariates were carried out in two stages. In the first stage, work, family and marital states were imputed by Corna *et al*,^25^ creating 20 imputed data sets. Then we augmented the data sets with imputation of missing information on the covariates and outcome using multiple imputations by chained equations.^32^ Records with imputed FI measures were excluded from the analysis phase. See the online supplementary file table S2 for a description of imputed and non-imputed samples at baseline.

10.1136/jech-2016-207887.supp2 supplementary table

All analyses were estimated using wave 1 weights for core sample members to minimise bias from differential non-response at baseline. Multilevel modelling was used to estimate growth curve models of frailty by employment histories (allowing for random intercepts and slopes) using a maximum likelihood algorithm. Minimally and fully adjusted models were estimated stratified by gender. Sampling weights were used in all models. Covariates that were associated with neither employment histories nor FI were excluded from the fully adjusted model (see table 1 and table S3 in supplementary file). The minimally adjusted models included age, age^2^ and wave (all time-varying). The fully adjusted model for women additionally included partnership, an interaction between partnership and age, drinking, IMD and non-pension wealth (time-varying), self-rated health during childhood, education and fertility history (time invariant). For men, the fully-adjusted models additionally included partnership, drinking, IMD and non-pension wealth (time varying), marital history, self-rated health during childhood and education (time invariant). All analyses were performed in STATA SE V.14.0.

10.1136/jech-2016-207887.supp3 supplementary table

## Results

### Baseline sample characteristics

As table 2 shows, women who were non-employed throughout (NET) tended to be older and to have a higher FI, while those who took a short career break before returning to part-time work (SCB) were more likely to have a lower FI. Men who had been working full-time throughout (FTT) were more likely to be older, while those who experienced an early exit from paid work (FTE49) were younger on average. Men in the FTE49 group also had a higher mean FI, while those with FTT histories or who were late starters with an early exit (LSE60) tended to have lower FI values.

**Table 2:** Sample characteristics (mean/%) by gender-specific categories of lifetime employment histories*

Measure	Females (N=2765)							Males (N=1621)						
	FTT	NET	WA	LCB	SCB	FC→FT	FT→PT	p Value	FTT	NET	FTE49	FTE60	LSE60	p Value
Age														
Mean	70.76	73.04	69.03	72.00	69.29	69.74	69.07	0.001	72.45	70.51	68.89	70.02	69.77	<0.001
Education														
No qualification	21.92	26.72	5.79	13.70	13.27	13.39	5.22	0.048	51.04	3.88	11.67	32.71	0.70	<0.001
Further qualification	43.71	13.55	5.18	9.01	5.37	21.44	1.74		29.34	3.48	4.97	21.35	40.86	
Father's social class														
Non-manual	26.89	27.80	5.31	12.02	8.68	16.77	2.52	0.031	45.90	3.71	7.38	25.66	17.34	0.001
Manual	24.65	23.38	6.12	13.51	12.89	15.45	4.00		50.73	4.50	9.84	31.36	3.57	
Unemployment	27.37	22.40	4.18	15.40	10.02	16.11	4.52		41.49	4.03	15.51	33.09	5.89	
Social class														
Professional	50.96	12.31	2.42	10.88	8.42	11.81	3.20	0.117	40.17	4.17	6.15	20.77	28.73	<0.001
Unskilled manual	12.92	32.22	7.54	15.87	16.22	5.98	9.24		52.38	1.96	18.87	26.79	0.00	
Marital history														
Never married	66.82	18.00	2.09	3.30	2.57	5.54	1.66	<0.001	43.63	7.02	18.56	28.84	6.95	0.998
Long-term married	20.36	25.98	6.18	14.76	12.94	16.10	3.68		49.76	3.78	7.55	31.09	7.83	
Marriage ends early	31.42	19.35	6.05	9.06	5.30	24.62	4.19		41.29	6.83	25.69	19.47	6.72	
Marriage ends late	23.68	28.50	3.96	10.16	7.69	22.57	3.44		-	-	-	-	-	
Fertility history														
No children	69.75	17.98	1.73	2.29	4.63	1.60	2.03	<0.001	50.51	6.19	12.69	23.22	7.39	0.370
One child early	29.63	20.19	7.24	8.51	12.82	17.45	4.16		44.59	2.66	9.63	41.72	1.40	
One child later	26.70	29.64	2.19	26.32	7.52	6.59	1.04		50.67	3.72	8.09	29.41	8.11	
Children early	14.52	23.26	7.79	12.09	14.11	23.06	5.17		48.39	4.85	6.40	33.49	6.86	
Children later	15.19	37.50	3.87	24.34	6.58	10.56	2.95		45.85	3.95	7.10	26.08	17.02	
Early large family	16.25	27.26	6.95	12.01	13.53	20.50	3.51		48.96	2.97	15.48	27.01	5.57	
Smoking status														
Non-smoker	25.31	24.55	5.43	14.00	11.08	16.37	3.26	0.31	49.68	3.85	8.01	30.16	8.31	0.942
Smoker	27.89	27.06	7.88	6.35	12.51	12.77	5.53		41.96	7.10	20.36	27.39	3.19	
Drinking														
Mean	98.34	86.10	100.16	99.58	96.87	107.57	87.01	0.06	131.67	127.91	133.61	149.98	232.75	<0.001
IMD														
0	26.43	22.26	5.67	13.32	11.88	16.31	4.13	0.60	48.73	4.48	7.95	30.70	8.14	0.657
9	40.23	28.99	1.76	13.02	0.59	14.20	1.18		4.35	0.00	95.65	0.00	0.00	
Non-pension wealth														
Highest	22.76	26.34	6.21	13.04	10.60	17.62	3.44	0.006	40.92	3.88	6.27	28.53	20.40	0.034
Lowest	27.32	29.35	6.42	12.63	8.93	11.81	3.54		48.60	3.98	21.64	25.31	0.48	
Partnership														
Not alone	20.91	23.33	6.30	14.18	14.19	17.79	3.29	<0.001	49.90	4.00	8.20	29.25	8.66	0.753
Alone	30.99	26.58	5.03	11.90	7.86	13.84	3.79		45.35	4.96	13.33	31.53	4.82	
Self-rated health during childhood														
Poor	23.83	28.76	9.08	14.91	11.25	7.96	4.21	0.709	58.86	4.43	7.28	23.71	5.73	0.258
Fair	28.35	27.17	5.26	11.88	11.09	13.41	2.84		41.95	3.05	12.51	35.11	7.37	
Good	24.47	26.19	5.61	10.67	10.68	18.10	4.28		47.39	4.36	9.00	32.24	7.00	
Very good	24.52	25.57	6.11	13.52	10.82	15.82	3.64		51.85	4.51	8.91	28.87	5.85	
Excellent	27.31	21.48	4.89	14.68	12.28	16.49	2.87		47.45	4.19	9.80	28.26	10.31	
Frailty Index														
Mean	0.16	0.18	0.17	0.16	0.14	0.15	0.17	0.008	0.11	0.12	0.17	0.13	0.10	0.002

*Women's employment histories: FTT; NET; WA; LCB; SCB; FC→; FT→. Men's employment histories: FTT; NET; FTE49; FTE60; LSE60**.**

FC→FT family care to full-time work; FT→PT Full-time to part-time work; FTT full-time throughout; IMD, index of multiple deprivation; LCB long career break; NET not-employed throughout; SCB short career break; WA weak attachment.

Men's employment histories:

FTE49 full-time, exit at 49; FTE60 full-time, exit at 60; FTT full-time throughout; LSE60 late start, exit at 60; NET not-employed throughout.

### Multilevel models of frailty by employment histories

Table 3 presents the main effects for the relationship between employment histories and FI at baseline (age 60/65 years at wave 2). The coefficients for the interaction between employment histories and age give the effect of employment histories on rate of change in FI from age 60/65 years.

**Table 3:** Multilevel models of frailty by gender-specific categories of employment histories, regression coefficients b and 95% CIs

	Minimally adjusted*		Fully adjusted†	
	B	95% CI	b	95% CI
**Female (N=2765)**				
*Fixed effects*				
Age	0.003	(0.003 to 0.004)	0.003	(0.002 to 0.004)
Age^2^	0.00008	(0.00005 to 0.0001)	0.0001	(0.00006 to 0.0001)
Employment histories				
FTT	REF		REF	
NET	0.010	(−0.002 to 0.02)	0.005	(−0.006 to 0.016)
WA	0.006	(−0.012 to 0.024)	−0.002	(−0.019 to 0.016)
LCB	−0.007	(−0.019 to 0.006)	−0.007	(−0.019 to 0.006)
SCB	−0.017^*^	(−0.030 to −0.003)	−0.019^*^	(−0.032 to −0.006)
FC→FT	−0.008	(−0.020 to 0.004)	−0.011	(−0.023 to 0.001)
FT→PT	0.012	(−0.010 to 0.034)	0.005	(−0.016 to 0.025)
Age*employment histories				
FTT	REF		REF	
NET	−0.002^**^	(−0.003 to −0.0008)	−0.002^**^	(−0.003 to −0.007)
WA	−0.001	(−0.003 to 0.0007)	−0.001	(−0.003 to 0.0007)
LCB	0.0001	(−0.001 to 0.001)	0.00005	(−0.001 to 0.001)
SCB	0.000	(−0.001 to 0.001)	−0.0004	(−0.002 to 0.0009)
FC→FT	−0.0002	(−0.001 to −0.001)	−0.0003	(−0.001 to 0.0008)
FT→PT	−0.0003	(−0.003 to 0.002)	−0.0001	(−0.002 to 0.002)
Intercept	0.178	(0.160 to 0.175)	0.141	(0.123 to 0.158)
*Random effects*				
Level 1: residual	0.044	(0.043 to 0.045)	0.044	(0.043 to 0.045)
Level 2: intercept	0.079	(0.076 to 0.082)	0.071	(0.069 to 0.074)
Level 2: age	0.005	(0.004 to 0.005)	0.004	(0.004 to 0.005)
**Male (N=1621)**				
*Fixed effects*				
Age	0.003	(0.002 to 0.004)	0.002	(0.001 to 0.003)
Age^2^	0.0002	(0.0001 to 0.0002)	0.0001	(0.0001 to 0.0002)
Employment histories				
FTT	REF		REF	
NET	0.004	(−0.018 to 0.026)	0.003	(−0.018 to 0.023)
FTE49	0.043^**^	(0.029 to 0.058)	0.036^**^	(0.022 to 0.050)
FTE60	0.021^**^	(0.012 to 0.030)	0.021^**^	(0.012 to 0.029)
LSE60	−0.002	(−0.016 to 0.013)	0.011	(−0.006 to 0.027)
Age*employment histories				
FTT	REF		REF	
NET	−0.001	(−0.004 to 0.001)	−0.001	(−0.004 to 0.001)
FTE49	−0.003^*^	(−0.004 to −0.0009)	−0.002^*^	(−0.004 to −0.0007)
FTE60	−0.0009	(−0.002 to 0.0001)	−0.0008	(−0.002 to 0.0002)
LSE60	0.0009	(−0.0008 to 0.003)	0.0009	(−0.0007 to 0.003)
Intercept	0.117	(0.111 to 0.123)	0.095	(0.074 to 0.115)
*Random effects*				
Level 1: residual	0.039	(0.039 to 0.040)	0.039	(0.038 to 0.040)
Level 2: intercept	0.068	(0.065 to 0.071)	0.064	(0.061 to 0.067)
Level 2: age	0.005	(0.005 to 0.006)	0.005	(0.005 to 0.006)

*Minimally adjusted model, for both females and males are adjusted for wave, time varying age and age^2^.

†Fully adjusted model, adjusted for wave, age, age^2^, partnership, drinking, index of multiple deprivation and non-pension wealth, fertility history and self-rated health during childhood, education, interaction between partnership and age for females; adjusted for wave, age, age^2^, partnership, drinking, index of multiple deprivation and non-pension wealth, marital history and self-rated health during childhood, education for males.

*<0.05, **≤0.001

FC→FT family care to full-time work; FT→PT Full-time to part-time work; FTT, full-time throughout; FTE, full-time exit; IMD, index of multiple deprivation; LCB, long career breaks; LSE, late start, early exit; NET, non-employed throughout; SCB, short career break; WA, weak attachment.

For women in the minimally adjusted model, the mean intercept at 60 years was 0.178. Both the linear and quadratic age terms were positive and statistically significant, indicating accelerating health declines after this age. After adjustment, the intercept was attenuated but health still declined (increasing frailty) at the same rate. Women who had a SCB had significantly lower FI at 60 years than those working FTT in both the minimally and fully adjusted models (b −0.019, 95% CI −0.032 to −0.006). Women in the NET group had a relatively higher FI but a significantly slower rate of change in FI with age than those working FTT, in both minimally and fully adjusted models (b −0.002 95% CI −0.003 to −0.007).

For men in the minimally adjusted model, the mean intercept at age 65 years and age slopes were 0.117, 0.003 and 0.0002 respectively, attenuating to 0.095, 0.002 and 0.0001 in the fully adjusted model. Men who started work young and exited early (FTE49 and FTE60) had significantly higher FI at baseline than those working FTT in the minimally and full adjusted models (FTE49 b 0.036, 95% CI 0.022 to 0.050; FTE60 b 0.021, 95% CI 0.012 to 0.029). However, FTE49 men had significantly slower increases in FI on average than FTT men in minimally (b −0.003, 95% CI −0.004 to −0.0009) and fully adjusted models (b −0.002 95% CI=−0.004 to −0.0007).

### Frailty trajectories by employment histories

**Figure 1:**
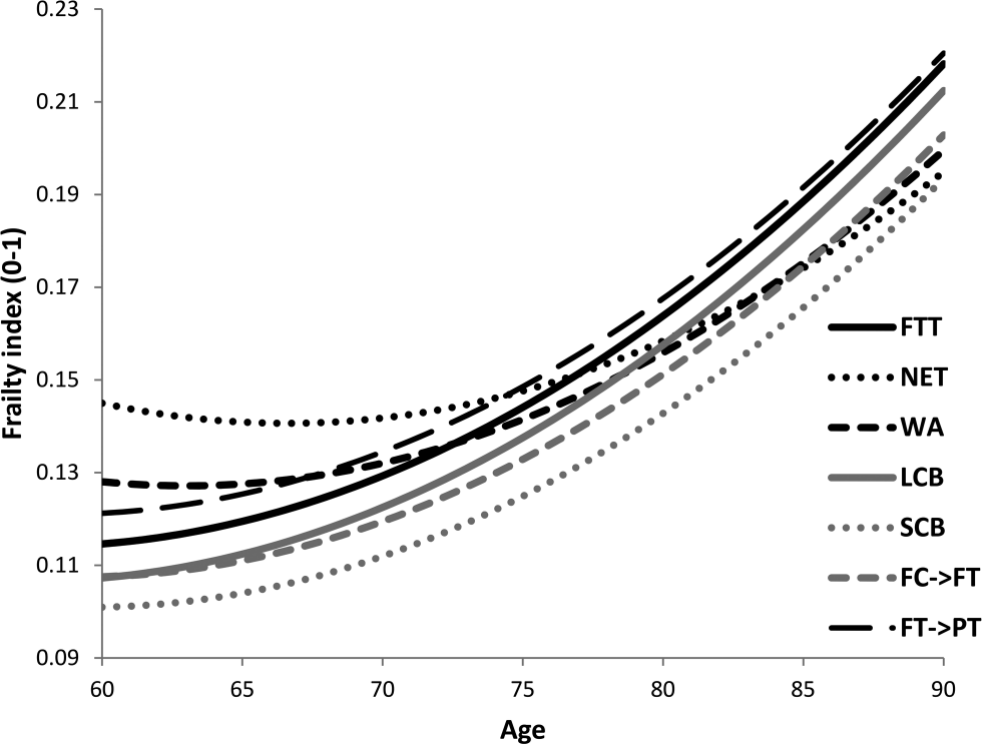
Estimated conditional trajectories^*^ of frailty for females by employment histories (N=2765). ^*^ For a woman with NVQ4/NVQ5/Degree or equivalent education qualifications, no drinking, married partnership, no children, excellent self-rated health in childhood, living in an area with IMD=0 (no deprivation) and the highest level of non-pension wealth. FC→FT family care to full-time work; FT→PT Full-time to part-time work; FTT, full-time throughout; IMD, index of multiple deprivation; LCB, long career breaks; NET, non-employed throughout; SCB, short career break; WA, weak attachment.

Conditional trajectories of frailty for women followed J-shapes with age (figure 1). With increasing age, all trajectories for the seven employment histories, especially FTT, LCB and FT→PT, tended to show a similarly high level of acceleration towards frailty. SCB women had the lowest levels of FI over the entire age-range in later life. NET women had the worst health profile at age 60 years but were projected to have the lowest FI levels in very old age.

**Figure 2:**
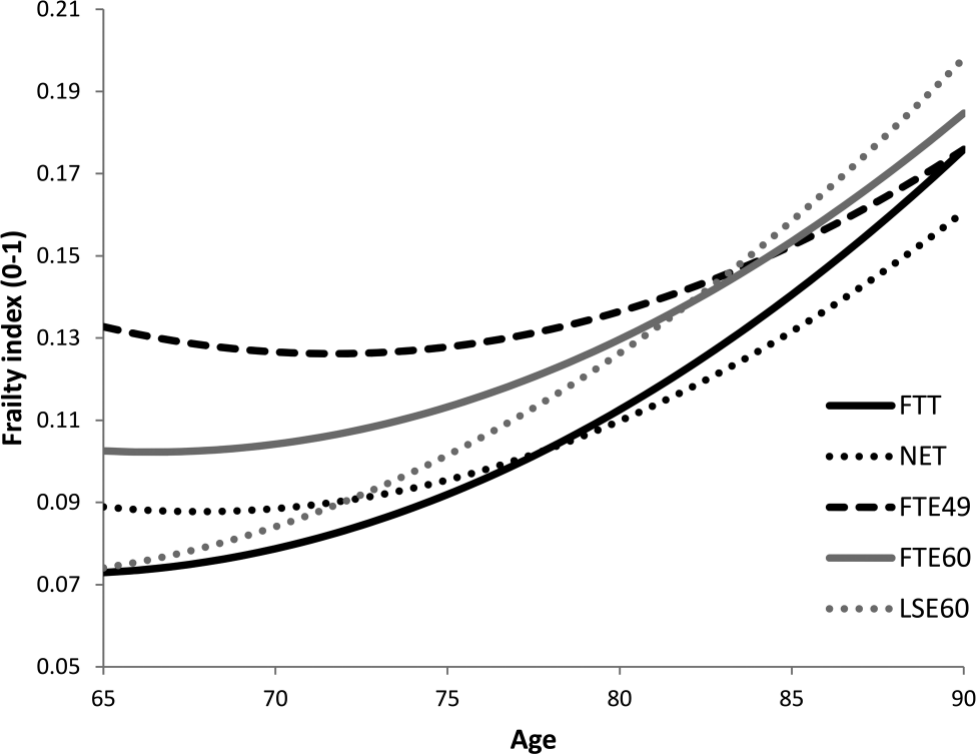
Estimated conditional trajectories^*^ of frailty for males by employment histories (N=1621). ^*^ Based on a man with NVQ4/NVQ5/Degree or equivalent education qualifications, no drinking, married partnership and martial histories, excellent self-rated health in childhood, living in area with IMD=0 (no deprivation) and the highest level of non-pension wealth. FTT, full-time throughout; FTE, full-time exit; IMD, index of multiple deprivation; LSE, late start, early exit; NET, non-employed throughout.

As expected, men on average had lower levels of FI than women (figure 2). The frailty trajectories for men showed greater variation across the various employment histories (despite the lack of significant differences in the coefficients in table 3) in comparison with those for women. Figure 2 also suggests that weaker lifetime labour market attachment is likely to be related to poor health during working age (NET, FTE49, FTE60 had highest FI levels at 65 years) and that early exit is related to slower decline thereafter.

## Discussion

Overall, frailty increased with age, accelerating after 65 and 70 years on average for women and men respectively. We found that experiencing distinct periods focused on work and family care may be advantageous for women in terms of lessening their risk of future frailty after 60 years. For men, being able to leave paid employment before 65 years if in poor health appears to slow down increases in frailty after retirement.

The findings showed that women who took a short break for family care and then undertook part-time work until 59 years (SCB) had lower FI at 60 years in comparison to those who were mostly in full-time work until 59 years (table 3 and figure 1). Thus, our findings seem to suggest the importance of work-life balance for women's later life health. Neither women who experienced long career breaks (LCB) or weak labour market attachment (WA) had as positive FI trajectories as those with histories combining family care with part-time work (SCB), suggesting that either weak labour market attachment or strong attachment at the expense of other roles, might both be detrimental for health. There is also a suggestion that women in the family care to full-time work (FC→FT) group followed a parallel (but higher) trajectory to those women in the SCB group, that is, with histories of family care and part-time work. If this result is also found in other studies, this may indicate that undertaking part-time work when, for example, children are young can have long-term positive benefits for women's health. Current employment policies encourage women's including mothers' participation in paid work. If we want to maintain the long-term health of today's generation of women, then our findings suggest the importance of flexible employment opportunities such as having flexible start and finish times according to children's age or working from home, for mothers who wish to combine work and family roles.

Almost half the women were in either the full-time throughout (FTT) or non-employed throughout (NET) life-time employment history groups. These two categories represented the two extremes of labour market attachment. The higher FI at age 60 years for NET women compared with FTT women is consistent with other research on the poor health of non-working women.^33^ More puzzling is NET women's slower frailty decline after age 60 years (figure 1), which was unaffected even when sociodemographic conditions and health behaviours in later life were taken into account. It is important to note that for the generation of women present in ELSA, full-time work throughout adulthood was relatively rare except for those who never-married (table 1). The FTT may have been a selected group and given rises in employment for all women including mothers its composition is likely to change in future.

Men with full-time work histories but who exited early from paid work at either 49 or 60 years were more likely to have higher FI at 65 years than those who kept working full-time until 65 years. The growth curve for FTE60 men in figure 2 describes a pattern with increasing level of FI after 65 years. However, a different picture emerges for those with FTE49 employment history: a flatter trajectory thereafter than men with other employment histories, consistent with their slower increase in frailty. Despite the possibility of selective attrition in this group, some other studies have commented on similar results: A study in France indicated that the burden of ill-health was substantially relieved by early retirement;^34^ A UK/US comparative study also found health improvements after taking a break from work among people who were in poor health.^35^ Therefore, being able to leave paid employment before 65 years, especially for those in poor health, seems to be able to slow down increases in frailty after retirement among men. Even though recent policies have introduced a range of measures to encourage older people to work longer, this research raises concerns that older people's health needs to be taken into consideration.

It is worth noting that men who started work later and exited around age 60 years (LSE60), had similarly low FI at 65 years as those who worked full-time from 16 to 64 years of age. Table 2 showed that nearly 41% of men with higher educational qualifications and 30% of men in the most advantaged social class were in the LSE60 group. The expectation was that they would have the best health, since their social advantage suggests that they would have experienced fewer health-harming environments over the life course, been more aware of health problems and found it easier to access to health services.^36^ ^37^ Yet their frailty increased more quickly after 65 years than those in the FTE49 and FTE60 groups. The small sample size of the LSE60 group (7.72%) may have resulted in a lack of power needed to detect different rates of decline in health for the LSE60 and FTT groups. Thus more evidence is needed to explore whether those in the LSE60 group actually experience more rapid increases in frailty in comparison to other groups.

This study has several limitations. First, self-reported health during childhood is the only available measure prior to working-age to control to for reverse causation; nevertheless, there may still be some residual confounding (eg, emotional or behavioural problems in childhood). Second, many items were self-reported when deriving the FI. This may have introduced reporting bias within participants. For example, people with pronounced weak work attachment might have over-reported frailty. Third, the samples of several of the employment history groups were small and therefore the analyses may have lacked power to detect significant relationships with frailty. For example, only 4% of men were in the NET group and the smallest mean difference in FI detectable in this group was relatively large (0.034). Nevertheless, the distributions of the employment histories are based on representative samples of older men and women in England. Finally, attrition by those in poor health may have introduced bias into the estimated growth curves although the multilevel models used all available data under a missing at random assumption.

In conclusion, this study's findings suggest that women benefit from histories reflecting work and family care. Experiencing distinct periods focusing on work and family care may be advantageous for women in terms of lessening the risk of future frailty after 60 years. For men, leaving the labour market early, possibly in response to poor health, appears to prevent increases in frailty following retirement. The findings above may help develop more efficient strategies to prevent frailty among older people. Improvements to recent employment policies such as extending working lives and providing flexible working options, could offer a healthier prospect for older people in the UK.

**What is already known on this subject**

- Frailty is a common clinical syndrome among the older adults, which leads to numerous poor health outcomes such as morbidity, incident disability or death. Working conditions like psychosocial hazards or economic inequalities, contribute to health inequalities. No previous research in England has explored the association between employment histories and frailty.

**What this study adds**

- We found experiencing distinct periods that focused on work and family care domains may be advantageous for women in terms of lessening their risk of future frailty after 60 years. Being able to leave paid employment before 65 years for these in poor health seems to be able to slow down increases in frailty after retirement among men.

## References

[1] Cracknell R The ageing population. Key Issues for the New Parliament 2010 2010:44–5.

[2] Department for Work & Pensions. 2010 to 2015 government policy: employment. Secondary 2010 to 2015 government policy: employment 8 May 2015 2013. https://www.gov.uk/government/publications/2010-to-2015-government-policy-employment/2010-to-2015-government-policy-employment#issue.

[3] World Health Organization. Patterns in disability and frailty in older adults: Evidence from SAGE 2010 http://www.who.int/healthinfo/11_Disability_frailty_Biritwum.pdf

[4] Xue QL The frailty syndrome: definition and natural history. Clin Geriatr Med 2011;27:1–15. 10.1016/j.cger.2010.08.009 21093718 PMC3028599

[5] Joosten E, Demuynck M, Detroyer E, et al Prevalence of frailty and its ability to predict in hospital delirium, falls, and 6-month mortality in hospitalized older patients. BMC Geriatr 2014;14:1 10.1186/1471-2318-14-1 24393272 PMC3905102

[6] Fried LP, Tangen CM, Walston J, et al Frailty in older adults: evidence for a phenotype. J Gerontol A Biol Sci Med Sci 2001;56:M146–56. 10.1093/gerona/56.3.M146 11253156

[7] Gale CR, Cooper C, Deary IJ, et al Psychological well-being and incident frailty in men and women: the English Longitudinal Study of Ageing. Psychol Med 2014;44:697–706. 10.1017/S0033291713001384 23822897 PMC3818135

[8] Syddall H, Roberts HC, Evandrou M, et al Prevalence and correlates of frailty among community-dwelling older men and women: findings from the Hertfordshire cohort study. Age Ageing 2010;39:197–203. 10.1093/ageing/afp204 20007127 PMC3546311

[9] Murray S Demography of death and dying. The University of Edinburgh: Primary Palliative Care Research Group. http://www.ed.ac.uk/files/atoms/files/demography_of_death_and_dying.pdf

[10] Rockwood K, Bergman H FRAILTY: a report from the 3rd Joint Workshop of IAGG/WHO/SFGG, Athens, January 2012. Can Geriatr J 2012;15:31–6. 10.5770/cgj.15.35 23259017 PMC3516241

[11] Rodríguez-Mañas L, Féart C, Mann G, et al Searching for an operational definition of frailty: a Delphi method based consensus statement: the frailty operative definition-consensus conference project. J Gerontol A Biol Sci Med Sci 2013;68:62–7. 10.1093/gerona/gls119 22511289 PMC3598366

[12] Kulminski AM, Ukraintseva SV, Kulminskaya IV, et al Cumulative deficits better characterize susceptibility to death in elderly people than phenotypic frailty: lessons from the Cardiovascular Health Study. J Am Geriatr Soc 2008;56:898–903. 10.1111/j.1532-5415.2008.01656.x 18363679 PMC2703425

[13] Marshall A, Nazroo J, Tampubolon G, et al Cohort differences in the levels and trajectories of frailty among older people in England. J Epidemiol Community Health 2015;69:316–21. 10.1136/jech-2014-204655 25646207 PMC4392235

[14] Hubbard RE, Lang IA, Llewellyn DJ, et al Frailty, body mass index, and abdominal obesity in older people. J Gerontol A Biol Sci Med Sci 2010;65:377–81. 10.1093/gerona/glp186 19942592

[15] Clark SC Work/family border theory: a new theory of work/family balance. Hum Relations 2000;53:747–70. 10.1177/0018726700536001

[16] Lewis J, Campbell M UK work/family balance policies and gender equality, 1997–2005. Soc Polit 2007;14:4–30. 10.1093/sp/jxm005

[17] Marmot M, Oldfield Z, Clemens S, et al English Longitudinal Study of Ageing: waves 0–6, 1998–2013. Secondary English Longitudinal Study of Ageing: waves 0–6, 1998–2013 2015 10.5255/UKDA-SN-5050-10

[18] Song X, Mitnitski A, Rockwood K Prevalence and 10-year outcomes of frailty in older adults in relation to deficit accumulation. J Am Geriatr Soc 2010; 58:681–7. 10.1111/j.1532-5415.2010.02764.x 20345864

[19] García-González JJ, Garcia-Peña C, Franco-Marina F, et al A frailty index to predict the mortality risk in a population of senior Mexican adults. BMC Geriatr 2009;9:47 10.1186/1471-2318-9-47 19887005 PMC2776593

[20] Rockwood K, Andrew M, Mitnitski A A comparison of two approaches to measuring frailty in elderly people. J Gerontol A Biol Sci Med Sci 2007;62:738–43. 10.1093/gerona/62.7.738 17634321

[21] Rockwood K, Fox RA, Stolee P, et al Frailty in elderly people: an evolving concept. CMAJ 1994;150:489–95. 8313261 PMC1486322

[22] Halpin B. Multiple Imputation for Life-Course Sequence Data 2012 http://teaching.sociology.ul.ie/seqanal/shortptex.pdf

[23] Abbott A, Tsay A Sequence analysis and optimal matching methods in sociology: review and prospect. Sociological Methods Res 2000;29:3–33. 10.1177/0049124100029001001

[24] Wiggins RD, Erzberger C, Hyde M, et al Optimal matching analysis using ideal types to describe the lifecourse: an illustration of how histories of work, partnerships and housing relate to quality of life in early old age. Int J Soc Res Methodol 2007;10:259–78. 10.1080/13645570701542025

[25] Corna LM, Platts LG, Worts D, et al A sequence analysis approach to modelling the work and family histories of older adults in the UK. Secondary A sequence analysis approach to modelling the work and family histories of older adults in the UK 2016 http://wherl.ac.uk/wp-content/uploads/2016/04/WHERL-Working-Paper-I.pdf

[26] Birnie K, Cooper R, Martin RM, et al Childhood socioeconomic position and objectively measured physical capability levels in adulthood: a systematic review and meta-analysis. PLoS ONE 2011;6:e15564 10.1371/journal.pone.0015564 21297868 PMC3027621

[27] Rice NE, Lang IA, Henley W, et al Common health predictors of early retirement: findings from the English Longitudinal Study of Ageing. Age Ageing 2011;40:54–61. 10.1093/ageing/afq153 21148324

[28] Kubo J, Goldstein BA, Cantley LF, et al Contribution of health status and prevalent chronic disease to individual risk for workplace injury in the manufacturing environment. Occup Environ Med 2014;71:159–66. 10.1136/oemed-2013-101653 24142977 PMC3932962

[29] Alvarado BE, Zunzunegui MV, Béland F, et al Life course social and health conditions linked to frailty in Latin American older men and women. J Gerontol A Biol Sci Med Sci 2008;63:1399–406. 10.1093/gerona/63.12.1399 19126855

[30] Lang IA, Hubbard RE, Andrew MK, et al Neighborhood deprivation, individual socioeconomic status, and frailty in older adults. J Am Geriatr Soc 2009;57:1776–80. 10.1111/j.1532-5415.2009.02480.x 19754500

[31] Sirven N On the socio-economic determinants of frailty: findings from panel and retrospective data from SHARE. Irdes 2012:18–22. http://www.irdes.fr/EspaceAnglais/Publications/WorkingPapers/DT52SocioEconomicDeterminantsFrailty.pdf

[32] Azur MJ, Stuart EA, Frangakis C, et al Multiple imputation by chained equations: what is it and how does it work? Int. J. Methods Psychiatr. Res. 2010; 20:40–9. 10.1002/mpr.329 PMC3074241 21499542

[33] Stone J, Evandrou M, Falkingham J, et al Women's economic activity trajectories over the life course: implications for the self-rated health of women aged 64+in England. J Epidemiol Community Health 2015;69:873–9. 10.1136/jech-2014-204777 25888594

[34] Westerlund H, Kivimäki M, Singh-Manoux A, et al Self-rated health before and after retirement in France (GAZEL): a cohort study. Lancet 2009;374: 1889–96. 10.1016/S0140-6736(09)61570-1 19897238

[35] Sacker A, Wiggins RD, Bartley M, et al Self-rated health trajectories in the United States and the United Kingdom: a comparative study. Am J Public Health 2007;97:812–8. 10.2105/AJPH.2006.092320 17395850 PMC1854880

[36] Bartley M Models of aetiological pathways, III: the material model. Health inequality: an introduction to theories, concepts and methods. Cambridge: Polity Press, 2004:90–102.

[37] Marmot MG Status syndrome: a challenge to medicine. JAMA 2006;295:1304–7 10.1001/jama.295.11.1304 16537740

