# Supplementary material for: Relationship between employment histories and frailty trajectories in later life: evidence from the English Longitudinal Study of Ageing
Source: J Epidemiol Community Health. 2016 Dec 2;71(5):439–45. doi: 10.1136/jech-2016-207887 (PMC5484034; doi:10.1136/jech-2016-207887)
Supplement: supplementary table [file jech-2016-207887supp001.pdf]

Table S1. Deficits included in calculating FI and their coding*

Description	Values (1 = a deficit, 0 = no deficit)	
	No	Yes
Difficulty walking 100 yards	0	1
Difficulty sitting 2 hours	0	1
Difficulty getting up from a chair after sitting long periods	0	1
Difficulty climbing several flights of stairs without resting	0	1
Difficulty climbing one flight of stairs without resting	0	1
Difficulty stooping, kneeling, or crouching	0	1
Difficulty reaching or extending arms above shoulder level	0	1
Difficulty pulling or pushing large objects like a living room chair	0	1
Difficulty lifting or carrying weights over 10 pounds, like a heavy bag	0	1
Difficulty picking up a 5p coin from a table	0	1
Difficulty dressing, including putting on shoes and socks	0	1
Difficulty walking across a room	0	1
Difficulty bathing or showering	0	1
Difficulty eating, such as cutting up your food	0	1
Difficulty getting in or out of bed	0	1
Difficulty using the toilet, including getting up or down	0	1
Difficulty using a map to figure out how to get around in a strange place	0	1
Difficulty preparing a hot meal	0	1
Difficulty shopping for groceries	0	1
Difficulty making telephone calls	0	1
Difficulty taking medications	0	1
Difficulty managing money, (e.g. paying bills and keeping track of expenses)	0	1
Difficulty doing work around the house or garden	0	1
Whether respondent has felt depressed much of the time during past week	0	1
Whether respondent felt everything they did during the past week was an effort	0	1
Whether respondent felt their sleep was restless during the past week	0	1
Whether respondent was happy much of the time during the past week	1	0
Whether respondent felt lonely much of the time during the past week	0	1
Whether respondent enjoyed life much of the time during the past week	1	0

Whether respondent felt sad much of the time during the past week		0			1	
Whether respondent could not get going much of the time during the past week		0			1	
High blood pressure or hypertension (self-reported)		0			1	
Angina (self-reported)		0			1	
Heart attack (including myocardial infarction or coronary thrombosis) (self-reported)		0			1	
Congestive heart failure (self-reported)		0			1	
An abnormal heart rhythm (self-reported)		0			1	
Diabetes or high blood sugar (self-reported)		0			1	
A stroke (cerebral vascular disease) (self-reported)		0			1	
Chronic lung disease such as chronic bronchitis or emphysema (self-reported)		0			1	
Asthma (self-reported)		0			1	
Arthritis (including osteoarthritis, or rheumatism) (self-reported)		0			1	
Osteoporosis, sometimes called thin or brittle bones (self-reported)		0			1	
Cancer or a malignant tumour (excluding minor skin cancers) (self-reported)		0			1	
Parkinson's disease (self-reported)		0			1	
Any emotional, nervous or psychiatric problems (self-reported)		0			1	
Alzheimer's disease (self-reported)		0			1	
Dementia, organic brain syndrome, senility or any other serious memory impairment (self-reported)		0			1	
Whether respondent has fallen down at all /last year /last 2years		0			1	
Whether respondent has fractured hip ever /in last 2 years		0			1	
Whether respondent has had joint replacement ever		0			1	
Whether respondent has had pain whilst walking		0			1	
Identify today's date: day of month		1			0	
Identify today's date: month		1			0	
Identify today's date: year		1			0	
Identify the day of the week		1			0	
	1st	2nd		3rd	4th	5th
Delayed word recall (sample organised into quintiles)	0	0.25		0.5	0.75	1
Immediate word recall (sample organised into quartiles)	0	0.3		0.6	1	-
	Excellent	Very good	Good	Fair	Poor	Blind

Self-reported eyesight (while using lenses, if appropriate)	0	0.2	0.4	0.6	0.8	1
Self-reported hearing (while using hearing aid if appropriate)	0	0.25	0.5	0.75	1	-
Self-reported general health	0	0.25	0.5	0.75	1	-

*Adapted from Marshall et al [13].
