# Supplementary material for: Relationship between employment histories and frailty trajectories in later life: evidence from the English Longitudinal Study of Ageing
Source: J Epidemiol Community Health. 2016 Dec 2;71(5):439–45. doi: 10.1136/jech-2016-207887 (PMC5484034; doi:10.1136/jech-2016-207887)

Figure S1. Categories of employment histories in women

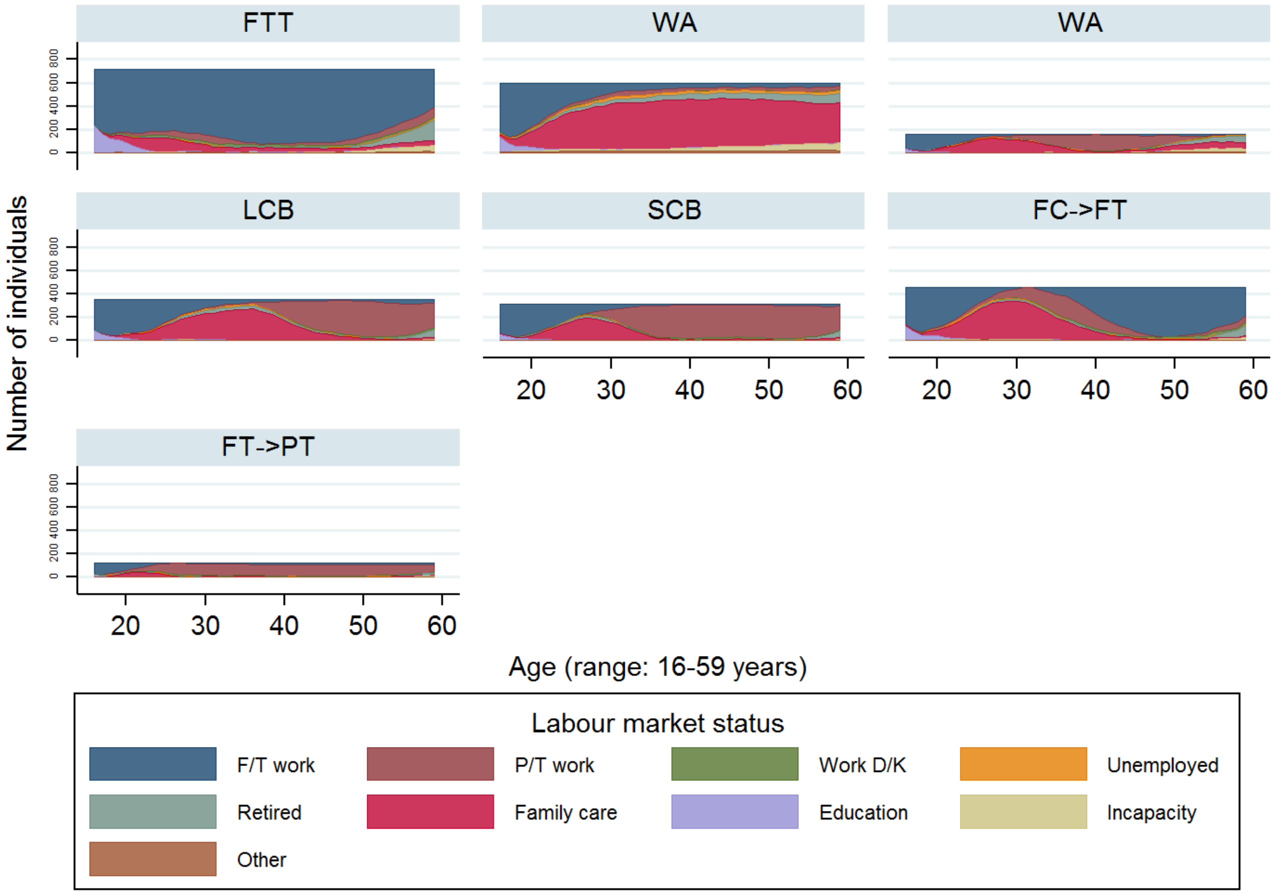

Figure S2. Categories of employment histories in men

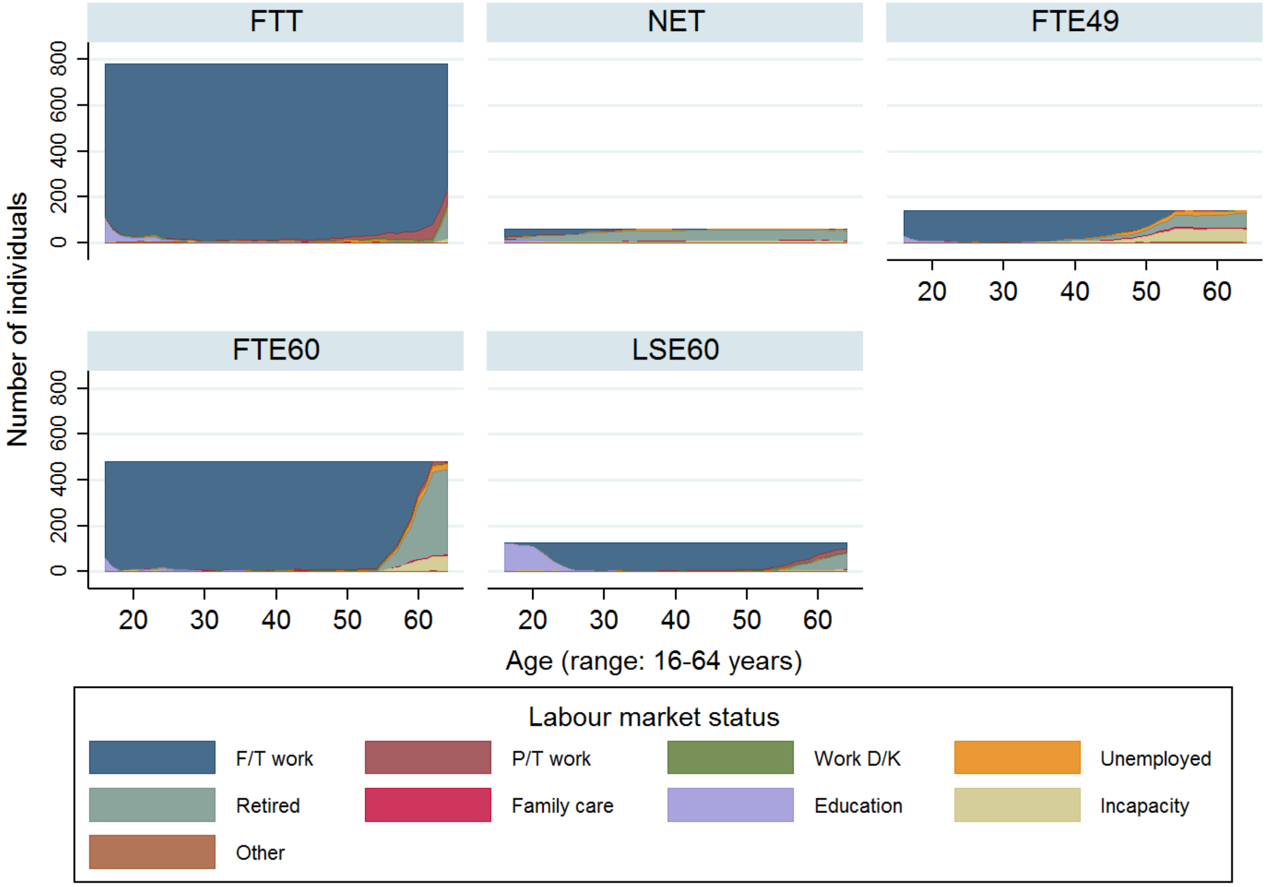

Supplement: supplementary figures [file jech-2016-207887supp002.pdf]
