# Supplementary material for: Relationship between employment histories and frailty trajectories in later life: evidence from the English Longitudinal Study of Ageing
Source: J Epidemiol Community Health. 2016 Dec 2;71(5):439–45. doi: 10.1136/jech-2016-207887 (PMC5484034; doi:10.1136/jech-2016-207887)
Supplement: supplementary table [file jech-2016-207887supp003.pdf]

Table S2. Description of imputed and non-imputed samples at baseline

	Female		Male	
	Imputed sample description	Non-imputed sample description	Imputed sample description	Non-imputed sample description
**Measure**	**%/Mean (95%CI)**	**%/Mean (95%CI)**	**%/Mean (95%CI)**	**%/Mean (95%CI)**
**Age**	70.47 (70.41-70.53)	70.50(70.43-70.56)	71.70 (71.63-71.77)	71.67 (71.60-71.74)
**Education %**				
No qualification	53.52 (53.11-53.93)	51.17 (50.64-51.69)	38.04 (37.52-38.56)	35.03 (34.46-35.62)
NVQ1/CSE other grade equivalent	1.87 (1.77-1.99)	1.97 (1.83-2.12)	10.39 (10.07-10.72)	10.11 (9.75-10.49)
NVQ2/GCE O Level equivalent	19.25 (18.93-19.58)	19.43 (19.02-19.84)	15.06 (14.68-15.45)	15.86 (15.42-16.31)
NVQ3/GCE A Level equivalent	4.97 (4.80-5.15)	5.14 (4.91-5.38)	6.58 (6.33-6.85)	6.63 (6.34-6.94)
Higher education below degree	11.83 (11.57-12.10)	12.49 (12.15-12.84)	14.44 (14.07-14.82)	15.70 (15.26-16.14)
NVQ4/NVQ5/Degree or equivalent	8.55 (8.32-8.78)	9.80 (9.50-10.12)	15.49 (15.11-15.88)	16.66 (16.21-17.12)
**Social class %**				
Professional	1.71 (1.61-1.82)	2.33 (2.18-2.49)	9.86 (9.55-10.18)	10.60 (10.23-10.98)
Managerial technical	26.11 (25.76-26.48)	28.39 (27.92-28.87)	32.86 (32.36-33.36)	34.30 (34.73-34.88)
Skilled-non-manual	38.31 (37.91-38.71)	36.34 (35.84-36.85)	10.23 (9.91-10.56)	10.44 (10.07-10.82)
Skilled manual	8.98 (8.75-9.22)	9.21 (8.91-9.51)	30.43 (29.94-30.92)	29.29 (28.74-29.85)
Semi-skilled manual	16.66 (16.36-16.97)	16.02 (15.64-16.41)	12.70 (12.35-13.06)	12.06 (11.66-12.46)
Unskilled manual	8.21 (7.99-8.44)	7.71 (7.44-7.99)	3.92 (3.72-4.13)	3.32 (3.11-3.54)
**Father's social class %**				
Non-manual	34.48 (34.09-34.87)	35.15 (34.65-35.65)	29.58 (29.10-30.07)	30.82 (30.26-31.39)
Manual	58.72 (58.32-59.13)	58.34 (57.82-58.85)	64.06 (63.55-64.57)	62.70 (62.11-63.29)
Unemployment	6.80 (6.60-7.01)	6.52 (6.26-6.78)	6.36 (6.10-6.62)	6.47 (6.18-6.78)
**Marital history %**				
Never married	9.47 (9.24-9.72)	9.44 (9.14-9.75)	9.48 (9.18-9.80)	9.39 (9.04-9.75)
Long-term married	78.79 (78.45-79.12)	79.62 (79.19-80.04)	85.91 (85.53-86.27)	86.72 (86.30-87.13)
Married ends early	8.00 (7.78-8.22)	7.29 (7.03-7.57)	4.61 (4.39-4.84)	3.89 (3.66-4.13)
Married ends late	3.74 (3.58-3.90)	3.65 (3.46-3.85)	-	-
**Fertility history %**				
No children	12.96 (12.68-13.23)	13.21 (12.86-13.57)	14.95 (14.57-15.33)	14.80 (14.38-15.24)
One child early	11.65 (11.39-11.92)	11.00 (10.68-11.33)	7.86 (7.58-8.15)	7.77 (7.45-8.10)
One child later	14.55 (14.26-14.84)	13.87 (13.51-14.23)	23.11 (22.67-23.56)	22.57 (22.07-23.08)

	Female		Male	
	Imputed sample description	Non-imputed sample description	Imputed sample description	Non-imputed sample description
**Measure**	**%/Mean (95%CI)**	**%/Mean (95%CI)**	**%/Mean (95%CI)**	**%/Mean (95%CI)**
Children early	32.85 (32.46-33.23)	32.82 (32.33-33.31)	27.75 (27.27-28.23)	28.32 (27.77-28.67)
Children later	4.27 (4.11-4.44)	4.12 (3.92-4.34)	10.96 (10.63-11.30)	10.52 (10.15-10.90)
Early large family	23.72 (23.38-24.07)	24.99 (24.54-25.44)	15.38 (15.00-15.77)	16.02 (15.58-16.47)
**Smoking %**				
Non-smoker	87.59 (87.31-87.85)	88.58 (88.25-88.91)	88.16 (87.81-88.50)	87.86 (87.46-88.26)
Smoker	12.41 (12.15-12.69)	11.42 (11.09-11.75)	11.84 (11.50-12.19)	12.14 (11.74-12.54)
**Drinking**	88.72 (88.27-88.18)	94.44 (93.90-94.98)	138.35 (137.70-139.00)	137.45 (136.73-138.01)
**Index of multiple deprivation%**				
0	72.77 (72.40-73.13)	73.52 (73.06-73.98)	76.57 (76.12-77.02)	77.18 (76.67-77.69)
1	12.69 (12.42-12.96)	12.49 (12.15-12.84)	11.88 (11.54-12.23)	11.81 (11.43-12.21)
2	5.82 (5.63-6.02)	5.80 (5.56-6.05)	4.85 (4.63-5.08)	4.85 (4.60-5.12)
3	4.09 (3.93-4.25)	4.12 (3.92-4.34)	2.46 (2.30-2.63)	2.10 (1.94-2.29)
4	1.73 (1.62-1.84)	1.61 (1.49-1.75)	1.93 (1.79-2.08)	1.86 (1.70-2.03)
5	1.09 (1.00-1.17)	0.72 (0.63-0.81)	1.18 (1.07-1.31)	1.21 (1.09-1.35)
6	0.89 (0.82-0.97)	0.90 (0.80-1.00)	0.60 (0.52-0.69)	0.73 (0.63-0.84)
7	0.37 (0.32-0.42)	0.36 (0.30-0.43)	0.33 (0.27-0.40)	0.16 (0.12-0.22)
8	0.27 (0.23-0.32)	0.18 (0.14-0.23)	0.12 (0.09-0.17)	0.08 (0.05-0.12)
9	0.29 (0.25-0.34)	0.300.25-0.36)	0.07 (0.05-0.10)	0.00
**Non-pension wealth %**				
0	18.55 (18.24-18.87)	16.92 (16.53-17.31)	14.62 (14.24-15.00)	12.38 (11.98-12.79)
1	19.94 (19.61-20.27)	18.95 (18.54-19.36)	18.49 (18.08-18.90)	18.12 (17.66-18.60)
2	20.30 (19.97-20.63)	20.50 (20.08-20.93)	19.64 (19.22-20.07)	19.50 (19.02-19.99)
3	20.53 (20.20-20.86)	21.28 (20.85-21.71)	22.64 (22.19-23.08)	23.79 (23.27-24.31)
4	20.68 (20.35-21.02)	22.36 (21.92-22.79)	24.62 (24.17-25.08)	26.21 (25.68-27.75)
**Partnership %**				
Not alone	54.08 (53.67-54.49)	56.07 (55.55-56.59)	75.67 (75.20-76.12)	77.67 (77.16-78.17)
alone	45.92 (45.51-46.33)	43.93 (43.41-44.45)	24.33 (23.88-24.80)	22.33 (21.83-22.84)
**Self-rated health in childhood %**				

	Female		Male	
	**Imputed sample description**	**Non-imputed sample description**	**Imputed sample description**	**Non-imputed sample description**
**Measure**	**%/Mean (95%CI)**	**%/Mean (95%CI)**	**%/Mean (95%CI)**	**%/Mean (95%CI)**
Poor	4.47 (4.31-4.65)	4.42 (4.21-4.64)	2.87 (2.70-3.05)	2.91 (2.71-3.12)
Fair	8.95 (8.72-9.19)	9.56 (9.26-9.88)	8.78 (8.48-9.09)	8.82 (8.48-9.17)
Good	21.98 (21.64-22.32)	21.22 (20.79-21.65)	22.04 (21.60-22.48)	22.41 (21.91-22.92)
Very good	35.78 (35.39-36.18)	35.62 (35.13-36.13)	33.45 (32.95-33.96)	33.01 (32.44-33.59)
Excellent	28.82 (28.45-29.19)	29.17 (28.70-29.65)	32.86 (32.36-33.36)	32.85 (32.28-33.42)
