# Supplementary material for: Relationship between employment histories and frailty trajectories in later life: evidence from the English Longitudinal Study of Ageing
Source: J Epidemiol Community Health. 2016 Dec 2;71(5):439–45. doi: 10.1136/jech-2016-207887 (PMC5484034; doi:10.1136/jech-2016-207887)
Supplement: supplementary table [file jech-2016-207887supp004.pdf]

Table S3. Association between each covariate and FI at baseline

Measure	Women (N=2,253)			Men (N=1,621)		
	Coeff.	P-value	95% CI	Coeff.	P-value	95%CI
Age	.003	< **.001**	(.002, .003)	.002	< **.001**	(.001, .002)
Education	.007	< **.001**	(.004, .009)	.007	< **.001**	(.005, .009)
Social class	.008	< **.001**	(.004, .011)	.007	< **.001**	(.004, .011)
Father's social class	.008	**.029**	(.0008, .016)	.005	.175	(-.002, .013)
Marital history	.002	.604	(-.006, .011)	.016	**.023**	(.002, .029)
Fertility history	.0004	.747	(-.002, .003)	.002	.205	(-.001, .005)
Smoking	.023	**.003**	(.008, .038)	.020	**.008**	(.005, .034)
Drinking	-.0001	< **.001**	(-.0001, -.00006)	-.00007	< **.001**	(-.0001, -.00003)
Deprivation index	.018	< **.001**	(.015, .022)	.013	< **.001**	(.009, .018)
Non-pension wealth	-.017	< **.001**	(-.020, -.014)	-.013	< **.001**	(-.016, -.009)
Partnership	.036	< **.001**	(.027, .044)	.016	**.003**	(.005, .027)
Self-rated health in childhood	.017	< **.001**	(.013, .021)	.012	< **.001**	(.007, .016)
